# RSK1 SUMOylation is required for KSHV lytic replication

**DOI:** 10.1371/journal.ppat.1010123

**Published:** 2021-12-06

**Authors:** Zhenshan Liu, Chengrong Liu, Xin Wang, Wenwei Li, Jingfan Zhou, Peixian Dong, Maggie Z. X. Xiao, Chunxia Wang, Yucai Zhang, Joyce Fu, Fanxiu Zhu, Qiming Liang

**Affiliations:** 1 Research Center of Translational Medicine, Shanghai Children’s Hospital, Shanghai Jiao Tong University, Shanghai, China; 2 Shanghai Institute of Immunology, Department of Immunology and Microbiology, Shanghai Jiao Tong University School of Medicine, Shanghai, China; 3 Department of Biological Science, Florida State University, Tallahassee, Flordia, United States of America; 4 Faculty of Medicine, University of Alberta, Edmonton, Alberta, Canada; 5 Department of Critical Care Medicine, Shanghai Children’s Hospital, Shanghai Jiao Tong University, Shanghai, China; 6 Department of Statistics, University of California, Riverside, Riverside, California, United States of America; 7 Key Laboratory of Cell Differentiation and Apoptosis of Chinese Ministry of Education, Shanghai Jiao Tong University School of Medicine, Shanghai, China; 8 State Key Laboratory of Microbial Metabolism, Shanghai Jiao Tong University, Shanghai, China; National Cancer Institute, UNITED STATES

## Abstract

RSK1, a downstream kinase of the MAPK pathway, has been shown to regulate multiple cellular processes and is essential for lytic replication of a variety of viruses, including Kaposi’s sarcoma-associated herpesvirus (KSHV). Besides phosphorylation, it is not known whether other post-translational modifications play an important role in regulating RSK1 function. We demonstrate that RSK1 undergoes robust SUMOylation during KSHV lytic replication at lysine residues K110, K335, and K421. SUMO modification does not alter RSK1 activation and kinase activity upon KSHV ORF45 co-expression, but affects RSK1 downstream substrate phosphorylation. Compared to wild-type RSK1, the overall phosphorylation level of RxRxxS*/T* motif is significantly declined in RSK1^K110/335/421R^ expressing cells. Specifically, SUMOylation deficient RSK1 cannot efficiently phosphorylate eIF4B. Sequence analysis showed that eIF4B has one SUMO-interacting motif (SIM) between the amino acid position 166 and 170 (_166_IRVDV_170_), which mediates the association between eIF4B and RSK1 through SUMO-SIM interaction. These results indicate that SUMOylation regulates the phosphorylation of RSK1 downstream substrates, which is required for efficient KSHV lytic replication.

## Author summary

RSK1 is an essential celluar kinase for lytic replication of Kaposi’s sarcoma-associated herpesvirus (KSHV). Besides phosphorylation, it is not known whether other post-translational modifications play an important role in regulating RSK1 function. Here, we found that RSK1 is SUMOylated at lysine residues K110, K335, and K421, which is enhanced by KSHV lytic replication. RSK1 SUMOylation is required for effient KSHV lytic replication and mutations on its SUMOylation sites leads to reduced lytic gene expressions and impaired progeny virus production. Interestingly, SUMO modification does not alter RSK1 activation and kinase activity upon KSHV ORF45 co-expression, but affects RSK1 downstream substrate phosphorylation. Specifically, SUMOylation deficient RSK1 cannot efficiently phosphorylate eIF4B since SUMO-SIM interaction mediates the association between eIF4B and RSK1. These results indicate that SUMOylation regulates the phosphorylation of RSK1 downstream substrates, which is required for efficient KSHV lytic replication.

## Introuduction

Kaposi’s sarcoma-associated herpesvirus (KSHV), also known as human herpesvirus-8 (HHV-8), belongs to the γ-herpesvirus subfamily, which also includes Rhesus Macaque Rhadinovirus (RRV), Herpesvirus saimiri (HVS), Murine γ-herpesvirus 68 (MHV68), and Epstein-Barr virus (EBV) [1]. KSHV is the causative agent for Kaposi’s sarcoma (KS), the most common cancer in HIV-infected patients, and is also tightly linked to several lymphoproliferative malignancies, such as primary effusion lymphoma and multicentric Castleman’s disease [1,2]. Like other herpesviruses, KSHV has of two distinct life phases: latency and lytic replication. During latency, most KSHV-encoded genes are silenced and only a few latent genes and microRNAs are expressed. Lytic replication results in a cascade expression of viral genes (immediate-early, early, and late genes), culminating in virion assembly, and ultimately release of mature progeny viruses. A spontaneous switch from latent to lytic replication is common in a small percentage of cells within KS lesions and is thought to induce pro-inflammatory cytokines that promote KSHV tumorigenesis [1]. As an intracellular parasite, KSHV modulates host cellular signaling pathways to evade the host antiviral immune surveillance program and establish persistent infection. We previously demonstrated that the ORF45, a tegument as well as immediate-early protein of KSHV, interacts with and persistently activates cellular p90 ribosomal S6 kinases (RSKs), which is critical for KSHV lytic replication and progeny virus production [3,4]. KSHV ORF45 forms high molecular mass protein complexes with RSK and extracellular signal-regulated kinase (ERK). The complexes allow protection of RSK and ERK from dephosphorylation, resulting is persistent activation of both of them [5]. The ORF45-RSK axis is believed to promote transcription and translation of a subset of viral and cellular genes during KSHV lytic replication. This is achieved at least partially through the phosphorylation of c-Fos, an immediate early transcription factor, and eIF4B, a regulatory translation initiation factor, respectively, to enable efficient lytic replication [5,6].

RSKs, a family of serine-threonine kinases, are the direct targets and functional mediators of ERK1/ERK2. They are involved in the regulation of multiple cellular processes, such as gene expression, protein synthesis, cell cycle and growth, survival, proliferation, and differentiation [7,8]. Human genome encodes four RSK isoforms (RSK1-RSK4), which share 75–80% amino acid identity, and RSK1-RSK3 are detected in most human tissues [9]. Distinctive among kinases, RSKs consist of two functional kinase domains. The N-terminal kinase domain (NTKD) belongs to an AGC family of kinase (serine/threonine kinases defined based on the sequence similarity of catalytic domain found in PKA, PKG, and PKC enzymes) and C-terminal kinase domain (CTKD) is a calcium/calmodulin-dependent protein kinase. There is a linker region between the two domains and a C-terminal tail contains a regulatory docking site for ERK [10,11]. In response to growth factors, neurotransmitters, and hormones, mitogen-activated protein kinases (MAPKs), phosphoinositide-3-OH kinase (PI3K), and autophosphorylation coordinate the activation of RSKs [12]. Several critical phosphorylation sites, including Thr359, Ser363, Ser380, and Thr573, play essential roles in RSK activation [13]. However, whether other post-translational modifications are required for RSK activation or KSHV lytic replication remains elusive.

SUMOylation is a post-translational modification process, during which small ubiquitin-like modifiers (SUMOs) are covalently conjugated to lysine residues of the target protein substrates. Like ubiquitination, SUMOylation is a three-step enzymatic process involving activating enzyme E1 (SAE1/UBA2), conjugating enzyme E2 (Ubc9), and various E3 ligases. Sometimes, SUMO E3 ligases are dispensable for the target modifications [14]. Mammals encode at least three SUMO isoforms (SUMO1-3), with SUMO2 and SUMO3 sharing 96% identify. SUMO conjugation typically mediates selected protein-protein interaction through SUMO-SIM (SUMO-interaction motif) interaction, leading to subcellular localization changes or transcriptional repression of target proteins [14]. Multiple cellular kinases, such as AKT and PKC undergo SUMOylation modification, which impacts their kinase activity, stability, or substrate specificity [15–17]. Here, we showed that RSK1 is SUMOylated primarily at Lys^110^, Lys^335^, and Lys^421^ and that SUMOylation of these sites appears to be required for efficient KSHV lytic replication and progeny virus production. We demonstrated that SUMOylation does not affect RSK1 activation or kinase activity, but appearantly modulates its substrate phosphorylation. Specifically, we found that RSK1 SUMOylation is required for ORF45-induced eIF4B phosphorylation and revealed a SIM site in eIF4B that engages recruitment of RSK1 for its phophorylation. Thus, our findings revealed a molecular mechanism by which RSK1 SUMOylation regulates KSHV lytic replication.

## Results

### RSK1 SUMOylation is enhanced during KSHV lytic replication

**Fig 1:**
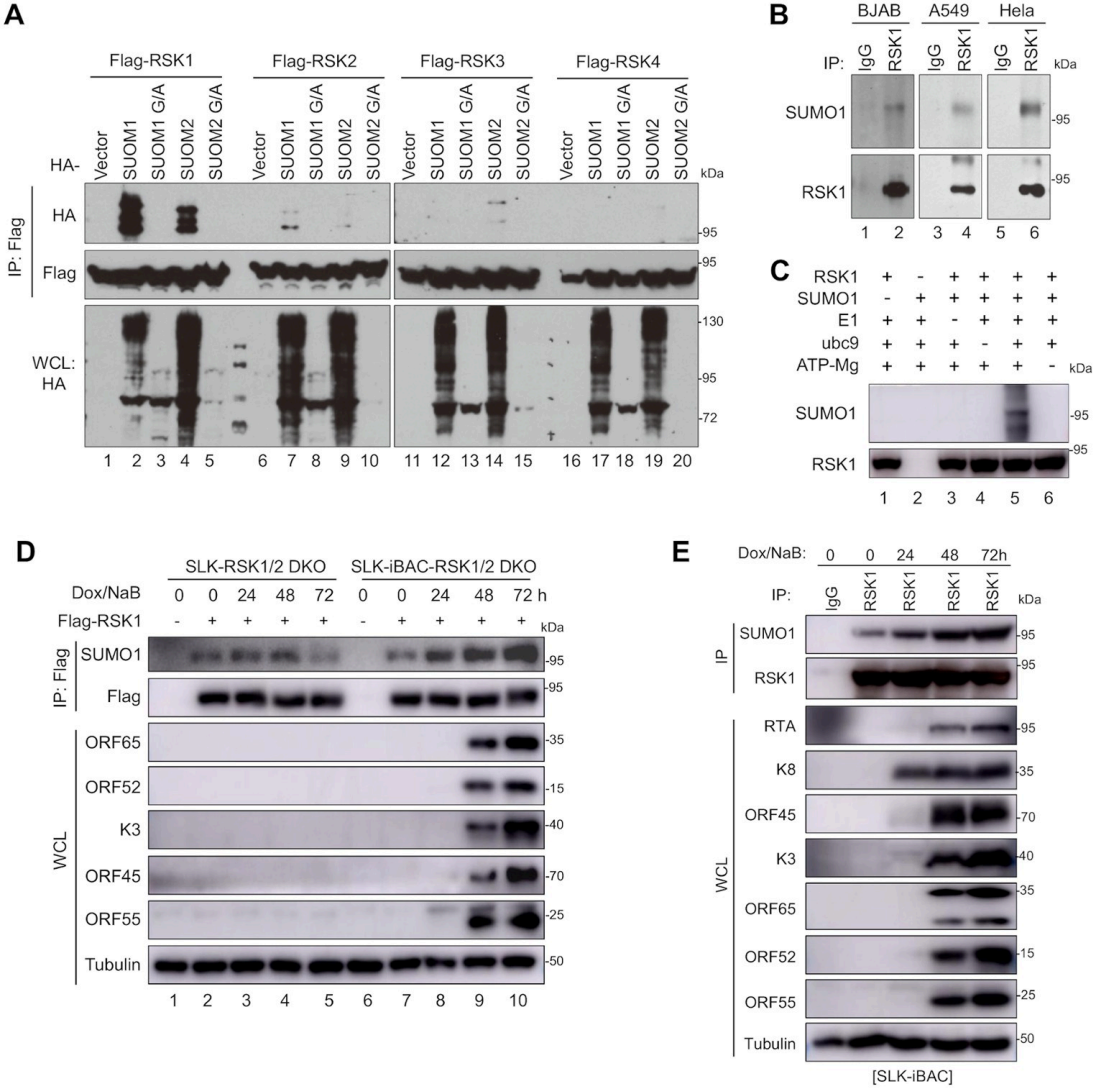
RSK1 undergoes SUMOylation during KSHV lytic replication. **(A)** RSK1 is highly SUMOylated in cells. HEK293T cells were co-transfected with indicated plasmids and cell lysates were subjected to IP and IB with indicated antibodies. **(B)** Endogenous RSK1 is SUMOylated. Endogenous RSK1 was immunoprecipitated with anti-RSK1 or IgG (control) antibodies under denature condition from lysates of BJAB, A549, and HeLa cells and the co-immunoprecipitated proteins were analyzed by IB with indicated antibodies. **(C)** RSK1 is SUMOylated *in vitro*. Recombinant RSK1 was incubated with SAE1/UBA2 (E1), Ubc9 (E2), SUMO1, and ATP/Mg in SUMO reaction buffer. The SUMOylation of RSK1 were detected by indicated antibodies. **(D)** RSK1 SUMOylation is increased during KSHV lytic replication. The SLK^RSK1/2 DKO^-vector, or SLK^RSK1/2 DKO^-Flag-RSK1, SLK-iBAC^RSK1/2 DKO^-vector, or SLK-iBAC^RSK1/2 DKO^-Flag-RSK1 cells were treated with doxycycline (2 μg/ml) and sodium butyrate (1 mM) to induce KSHV lytic replication. Cell lysates were harvested at indicated time points and subjected to IP under denature condition and IB with indicated antibodies. (**E**) The endogenous RSK1 SUMOylation is elevated during KSHV lytic replication. SLK-iBAC cells were treated with doxycycline (2 μg/ml) and sodium butyrate (1 mM) to induce KSHV lytic replication. Cell lysates were harvested at indicated time points and subject to IP with anti-RSK1 or control IgG under denature condition and IB with indicated antibodies.

SUMOylation of MEK (also known as MAPK kinase or MAP2K) has been shown to interfere with the specific docking interaction between MEK and ERK and thus negatively regulate ERK activation [18]. However the roles of SUMOylation in regulating other components of MAPK cascades remained unexplored. To determine whether SUMOylation regulates RSKs, we first determined whether RSKs undergo SUMOylation in cells. We expressed each Flag-tagged RSKs together with HA-tagged SUMO1, SUMO2, or their G/A mutants (SUMO1^G96A/G97A^ and SUMO2^G92A/G93A^) in HEK293T cells. SUMO G/A mutants cannot be ligated to lysine residues and served as a negative control for SUMOylation modification of RSKs. Flag-RSKs were immunoprecipitated under denature condition, resolved on SDS-PAGE, and immunoblotted with anti-Flag or anti-HA antibodies. The signal of SUMOylated RSKs migrated about 20 kDa from the unSUMOylated forms, and was abolished under SUMO G/A mutant conditions (lane 3 and lane 5) (Fig 1A). As shown in Fig 1A, RSK1 was strongly modified by both SUMO1 (lane 2) and SUMO2 (lane 4), the SUMOylation of RSK2 and RSK3 were much weaker than RSK1, while RSK4 SUMOylation was undetected in the same condition (Fig 1A), indicating different RSK isoforms are selectively modified by SUMOs. SUMO modification of endogenous RSK1 was also observed in BJAB, A549, and HeLa cells (Fig 1B). In addition, recombinant RSK1 is SUMOylated *in vitro* when mixed with SAE1/UBA2 (E1), Ubc9 (E2), SUMO1, and ATP/Mg (Fig 1C). These results showed that RSK1 is SUMOylated both *in vitro* and *in vivo*.

Next, we examined RSK1 SUMOylation levels over time during KSHV lytic replication. We modified BAC16 by replacing the GFP with TET3G under EF1α promoter and inserted seven tet response elements in the promoter of RTA to generate iBAC (GenBank accession number: OK358814) (S1 Fig), in which RTA expression is controlled by a Tet-On promoter on BACmid [19]. To aid detection of RSK1 SUMOylation, we stably expressed Flag-tagged RSK1 in SLK-iBAC cells with both RSK1 and RSK2 knockout by CRISPR-Cas9 (SLK^RSK1/2 DKO^-iBAC). SLK-iBAC cell entered lytic replication cycle upon doxycycline and sodium butyrate treatment as shown by immunoblots of several lytic genes in the time course (Fig 1D and 1E). RSK1 SUMOylation was detected in SLK^RSK1/2 DKO^-iBAC-Flag-RSK1 cells, and increased over time after induction of KSHV lytic replication (Fig 1D). The endogenous RSK1 SUMOylation was also elevated during KSHV lytic replication in SLK-iBAC cells (Fig 1E). These results demonstrated that RSK1 is SUMOylated during KSHV lytic replication, suggesting its potential role in regulating KSHV replication.

### Identification of RSK SUMOylation sites

**Fig 2:**
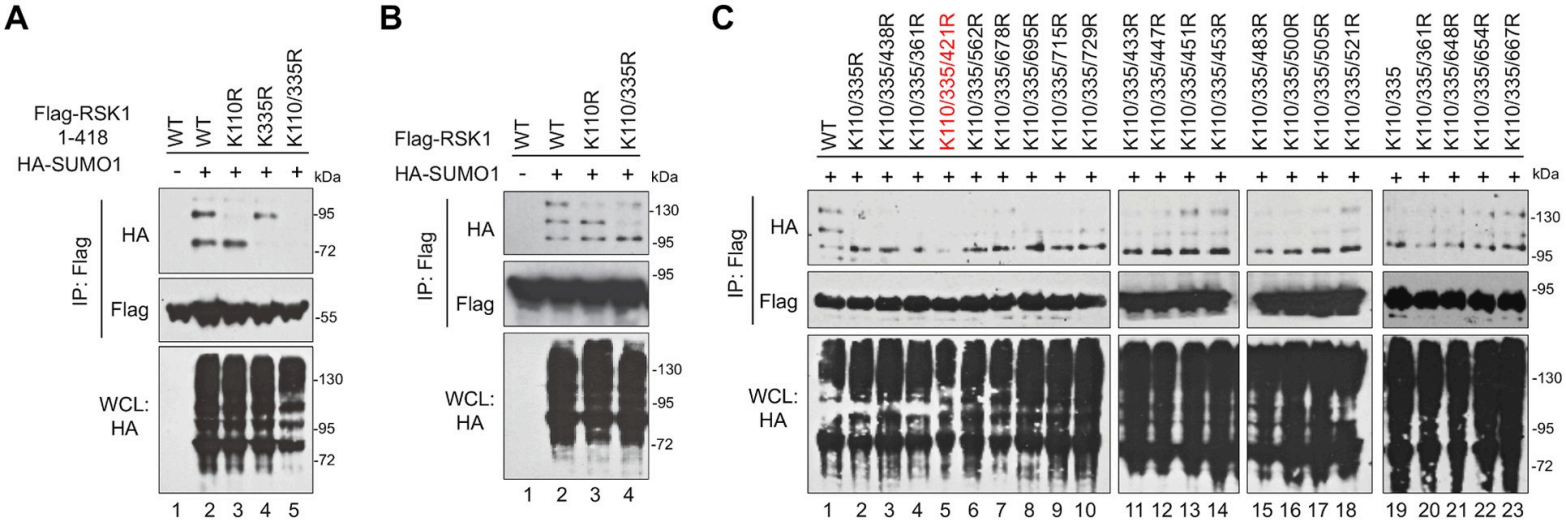
Identification of RSK1 SUMOylation sites. (**A**) Lys^110^ and Lys^335^ of RSK1 are two primary SUMOylation sites in RSK1 N-terminal fragments. HEK293T cells were co-transfected with indicated plasmids and cell lysates were subjected to IP and IB with indicated antibodies. (**B**) Lys^110^ and Lys^335^ are two SUMO sites of RSK1. Similar procedure as in Fig 2A. (**C**) Lys^110^ and Lys^335^, and Lys^421^ are three major SUMO sites of RSK1. Similar procedure as in Fig 2A.

Next, we sought to determine which sites of RSK1 are primarily modified by SUMOs. SUMO1 modification of RSK1 resulted in three prominent shifted bands (110~170 kDa) above the unmodified RSK1 (~90 kDa), suggesting at least three different lysine residues are primarily modified by SUMOs (Fig 1A). To locate the SUMOylation sites on RSK1, we truncated it into two fragments, RSK1^NT^ [amino acid (aa) 1–418] and RSK1^CT^ (aa 321–735). When co-expressed with SUMO1, RSK1^NT^ generated two additional bands above the unmodified form, indicating two major lysines are SUMOylated. Mutagenesis analysis revealed that Lys^110^ and Lys^335^ were two primary SUMOylation sites and mutating both of them to arginine abolished RSK1^NT^ SUMOylation (Fig 2A). Consistently, K110/335R double mutation in the full-length RSK1 reduced SUMOylation bands from three to only one, comparing lane 4 to 2 (Fig 2B). This result not only confirms that K110 and K335 are primary SUMOylation sites in aa1-418 region but also suggests an additional SUMO site located in the aa 418–735 region. Therefore, we generated more lysine to arginine mutations on the RSK1^K110/335R^ backbone to locate the third SUMOylation site. As shown in Fig 2C, additional mutation of Lys^421^ to arginine significantly inhibited RSK1^K110/335R^ SUMOylation (comparing lane 5 to 1), while mutations on other lysine sites, such as Lys^361^, Lys^438^, Lys^562^, Lys^678^, Lys^695^, Lys^715^, Lys^729^, Lys^433^, Lys^447^, Lys^451^, Lys^453^, Lys^483^, Lys^500^, Lys^505^, Lys^521^, Lys^648^, Lys^654^, and Lys^667^, had little or no effect (Fig 2C). These data demonstrated that Lys^110^, Lys^335^, and Lys^421^ of RSK1 are the three major sites that are modified by SUMO1.

### RSK1 SUMOylation is required for KSHV lytic replication and progeny virus production

**Fig 3:**
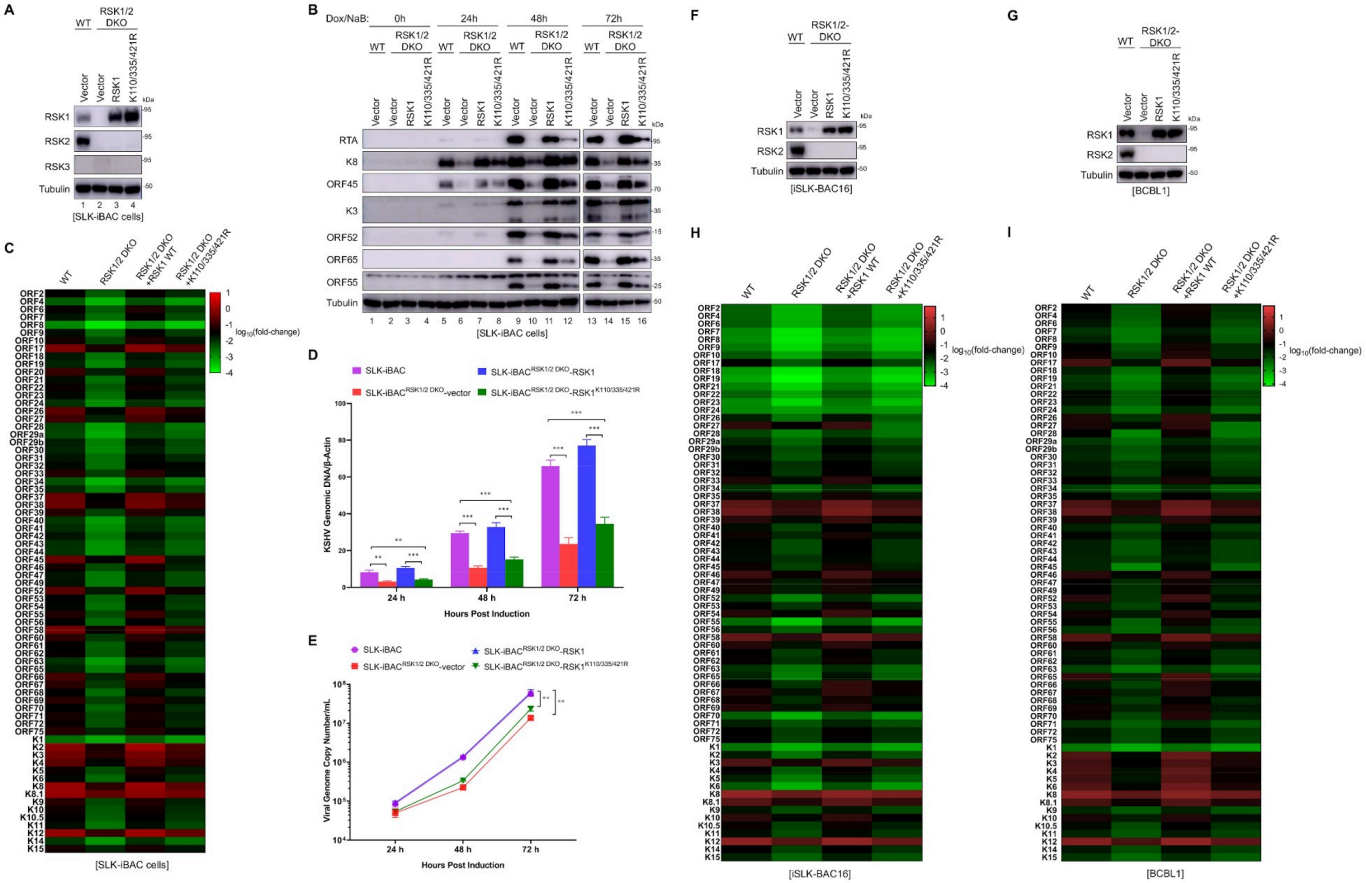
RSK1 SUMOylation is required for efficient KSHV lytic replication. (**A**) Generation of SLK-iBAC^RSK1/2 DKO^, SLK-iBAC^RSK1/2 DKO^-RSK1 or SLK-iBAC^RSK1/2 DKO^-RSK1^K110/335/421R^ cells by CRISPR-Cas9 and lentivirus-mediated stable expression. (**B-C**) RSK1 SUMOylation affects viral protein expression at both RNA and protein levels during KSHV lytic replication. Indicated cell lines were treated with doxycycline (2 μg/ml) and sodium butyrate (1 mM) to induce KSHV lytic replication. Cell lysates were collected at indicated time points and subjected to IB with indicated antibodies (B). Indicated SLK-iBAC cell lines were induced with doxycycline and sodium butyrate for 72 h. Total RNA was extracted, reverse-transcribed into cDNA, and used for KSHV whole-genome qPCR array analysis. The Δ*C*_*T*_ values for each primer set were calculated and converted to a heatmap using R (C). (**D-E**) RSK1 SUMOylation is required for efficient viral genomic DNA replication (D) as well as progeny virus production (E) during KSHV lytic replication. The cell lysates or culture medium containing progeny viruses were collected at indicated time point. Total DNA was isolated and viral genomic DNA was quantified by qPCR. (**F-G**) Generation of iSLK-BAC16^RSK1/2 DKO^, iSLK-BAC16^RSK1/2 DKO^-RSK1, iSLK-BAC16^RSK1/2 DKO^-RSK1^K110/335/421R^, BCBL1^RSK1/2 DKO^, BCBL1^RSK1/2 DKO^-RSK1, BCBL1^RSK1/2 DKO^-RSK1^K110/335/421R^ cells by CRISPR-Cas9 and lentivirus-mediated stable expression. (**H-I**) RSK1 SUMOylation affects viral protein expression during KSHV lytic replication. iSLK-BAC16 related cell lines (H) were treated with doxycycline (2 μg/ml) and sodium butyrate (1 mM) and BCBL1 related cell lines (I) were treated with TPA (20 ng/ml) to induce KSHV lytic replication. RNA samples were collected at 72 h post-induction and subjected to RT-qPCR analysis for overall KSHV gene expression. Heatmaps were generated using similar method as Fig 3C.

The activation of RSKs by ORF45 has been shown to be crucial for KSHV lytic replication [3,4], we next determined whether RSK1 SUMOylation plays a role in this process. RSK1 and RSK2 but not RSK3 are highly expressed in SLK-iBAC cells (Fig 3A) [19]. Genetic knockout of RSK1 and RSK2 by CRISPR-Cas9 dramatically reduced KSHV lytic gene expression at both the mRNA and protein levels, as well as viral DNA copy number, and progeny virus production (Fig 3B–3E). To determine whether SUMOylation of RSK1 is required for KSHV lytic replication, we complemented SLK^RSK1/2 DKO^-iBAC cells with RSK1^K110/335/421R^ or wild-type RSK1 (RSK1^WT^) and then induced lytic replication with doxycycline and sodium butyrate. We first examined viral protein expression by immunoblot. In SLK-iBAC^RSK1/2 DKO^ cells, the protein expression levels of immediate-early genes (RTA, K8, and ORF45), early genes (K3 and ORF52), and late genes (ORF55 and ORF65) were significantly rescued by RSK1^WT^ at 24, 48, and 72 h post-induction, to similar levels as wild-type SLK-iBAC cells (Fig 3B). However, RSK1^K110/335/421R^ SUMOylation deficient mutant only partially rescued these viral protein expressions (Fig 3B), indicating that RSK1 SUMOylation is required for efficient KSHV lytic replication. Next, to systematically compare KSHV viral gene expression profile between RSK1^WT^ and RSK1^K110/335/421R^ expressing cells, we examined viral mRNA levels by a genome-wide quantitative RT-PCR array at 72 h post lytic replication. As shown in Fig 3C, RSK1^WT^ dramatically elevated the overall mRNA levels of KSHV genes in SLK-iBAC^RSK1/2 DKO^ cells, while RSK1^K110/335/421R^ failed to rescue (Fig 3C). Consistently, viral DNA copy number was also significantly increased in SLK-iBAC^RSK1/2 DKO^-RSK1^WT^ but not in SLK-iBAC^RSK1/2DKO^-RSK1^K110/335/421R^ cells (Fig 3D). Last, we evaluated the progeny virus production by quantifying the viral genomic DNA levels in the culture supernatant. RSK1/2 double knockout dramatically reduced KSHV progeny virus production, and RSK1^WT^ but not its SUMOylation deficient mutant RSK1^K110/335/421R^ rescued progeny virus levels (Fig 3E). Furthermore, we knocked out both RSK1 and RSK2 in iSLK-BAC16 or BCBL1 cells and complemented wild-type RSK1 or RSK1^K110/335/421R^ by lentivirus (Fig 3F–3G). Consistently, RSK1^K110/335/421R^ cannot efficiently support KSHV lytic replication as RSK^WT^ in iSLK-BAC16^RSK1/2 DKO^ upon doxycycline and sodium butyrate treatment or in BCBL1^RSK1/2 DKO^ upon TPA treatment (Fig 3H and 3I). Together, these results demonstrated that RSK1 SUMOylation is required for efficient lytic replication and progeny virus production of KSHV.

### SUMOylation does not affect ORF45-induced RSK1 activation or kinase activity

**Fig 4:**
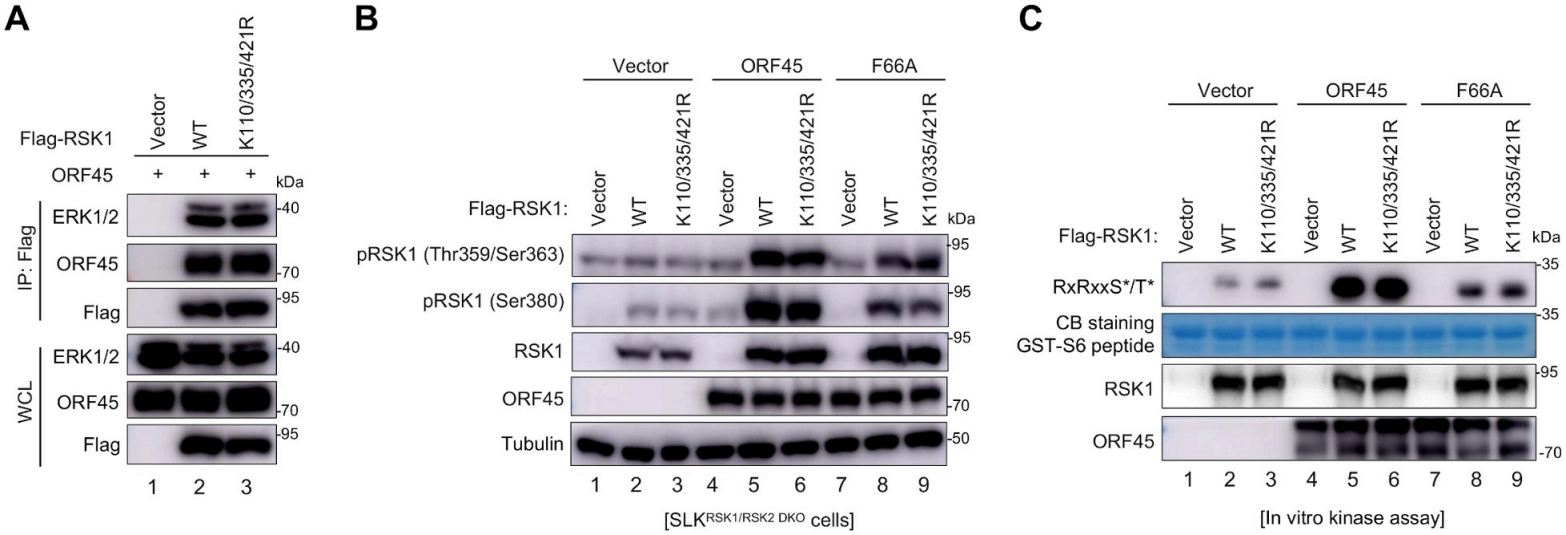
RSK1 SUMOylation does not alter its activation or kinase activity. (**A**) SUMOylation site mutations of RSK1 do not change ERK-RSK-ORF45 complex formation. HEK293T cells were co-transfected with indicated plasmids and cell lysates were subjected to IP and IB with indicated antibodies. (**B**) SUMOylation site mutations do not affect RSK1 phoshporylation by KSHV ORF45. SLK^RSK1/2 DKO^-vector, SLK^RSK1/2 DKO^-RSK1 and SLK^RSK1/2 DKO^-RSK1^K110/335/421R^ cells were infected with lentiviruses containing empty vector, ORF45^WT^ or ORF45^F66A^ as indicated. The cells were serum-starved for 24h before sample collection and cell lysates were analyzed by IB with indicated antibodies at 48 h post-transfection. (**C**) SUMOylation site mutations do not affect RSK1 kinase activity *in vitro* (Methods section). RSK1 kinase complexes with or without ORF45 were purified from HEK293A cells transfected with indicated plasmid. GST-S6 peptide was purified from *E*.*coli*. RSK1 (5 μl) were mixed with GST-S6 (2.5 μg) in kinase assay buffer at 30 °C for 30 min. The reaction was stopped by adding 2 x SDS-PAGE loading buffer, followed by IB analysis with indicated antibodies.

Next, we investigated how SUMOylation affects RSK1’s function during KSHV lytic replication. Since KSHV activates RSK1/2 through forming ERK-RSK-ORF45 complexes [5], we first determined whether SUMOylation affects ERK-RSK1-ORF45 association. Similar to RSK1^WT^, RSK1^K110/335/421R^ interacted with both ERK and ORF45 at similar level (Fig 4A), suggesting SUMOylation site mutations of RSK1 do not change ERK-RSK-ORF45 complex formation. Next, we examined whether SUMOylation regulates RSK1 activation by KSHV ORF45. We generated RSK1/2 double knockout SLK cells by CRISPR-Cas9, and stably complemented the expression of RSK1^WT^, RSK1^K110/335/421R^ SUMOylation deficient mutant, or vector control by lentiviruses. After lentivirus-mediated transient expression of wild-type ORF45 (ORF45^WT^, a potent activator), ORF45^F66A^ (a mutant that cannot bind to or activate RSKs) [3], or vector control, we evaluated the phosphorylation level of RSK1 at Thr359, Ser363, and Ser380, which indicate the activation level of RSK1 by the upstream signaling [9,13]. Consistent with our previous findings [3], KSHV ORF45^WT^ expression led to significantly increased phosphorylation of RSK1 at Thr359, Ser363, and Ser380, as compared to vector control, while ORF45^F66A^ did not (Fig 4B). Similar to RSK1^WT^, ORF45^WT^-mediated phosphorylation levels of Thr359, Ser363, or Ser380 were not affected by mutations on RSK1 SUMOylation sites (Fig 4B), indicating that SUMOylation has little or no effect on RSK1 activation by KSHV ORF45.

To further examine whether SUMOylation affects RSK1 kinase activity, we performed an *in vitro* kinase assay with RSK1 (purified from HEK293A cells) and recombinant GST-S6 peptide (purified from *E*.*coli*) containing the phosphorylation site by RSKs. S6 peptide (KEAKEKRQEQIAKRRRLSSLRASTSKSESSQK) [4] was cloned into pGEX-4T vector and recombinant GST-S6 peptide was purified from *E*.*coli* after isopropyl-β-D-thiogalactopyranoside (IPTG) induction. RSK1^WT^ or RSK1^K110/335/421R^ were immunoprecipitated by anti-Flag affinity beads from HEK293A cells transiently co-transfected with or without KSHV ORF45^WT^, ORF45^F66A^ (a mutant cannot activate RSK), or empty vector control. A specific phosphorylation antibody against RxRxxS*/T* motif was used to detect the phosphorylation of GST-S6 peptide by RSK1 [19]. As shown in Fig 4C, both wild-type and SUMOylation deficient RSK1 caused similar phosphorylation levels of GST-S6 peptide substrate, and ORF45^WT^ but not ORF45^F66A^ dramatically enhanced this phosphorylation (Fig 4C), indicating that SUMOylation does not affect RSK1 kinase activity *in vitro*. These results demonstrated that SUMOylation modification does not affect RSK1 activation or kinase activity by KSHV ORF45.

### RSK1 SUMOylation affects the phosphorylation levels of its downstream substrates in cells

**Fig 5:**
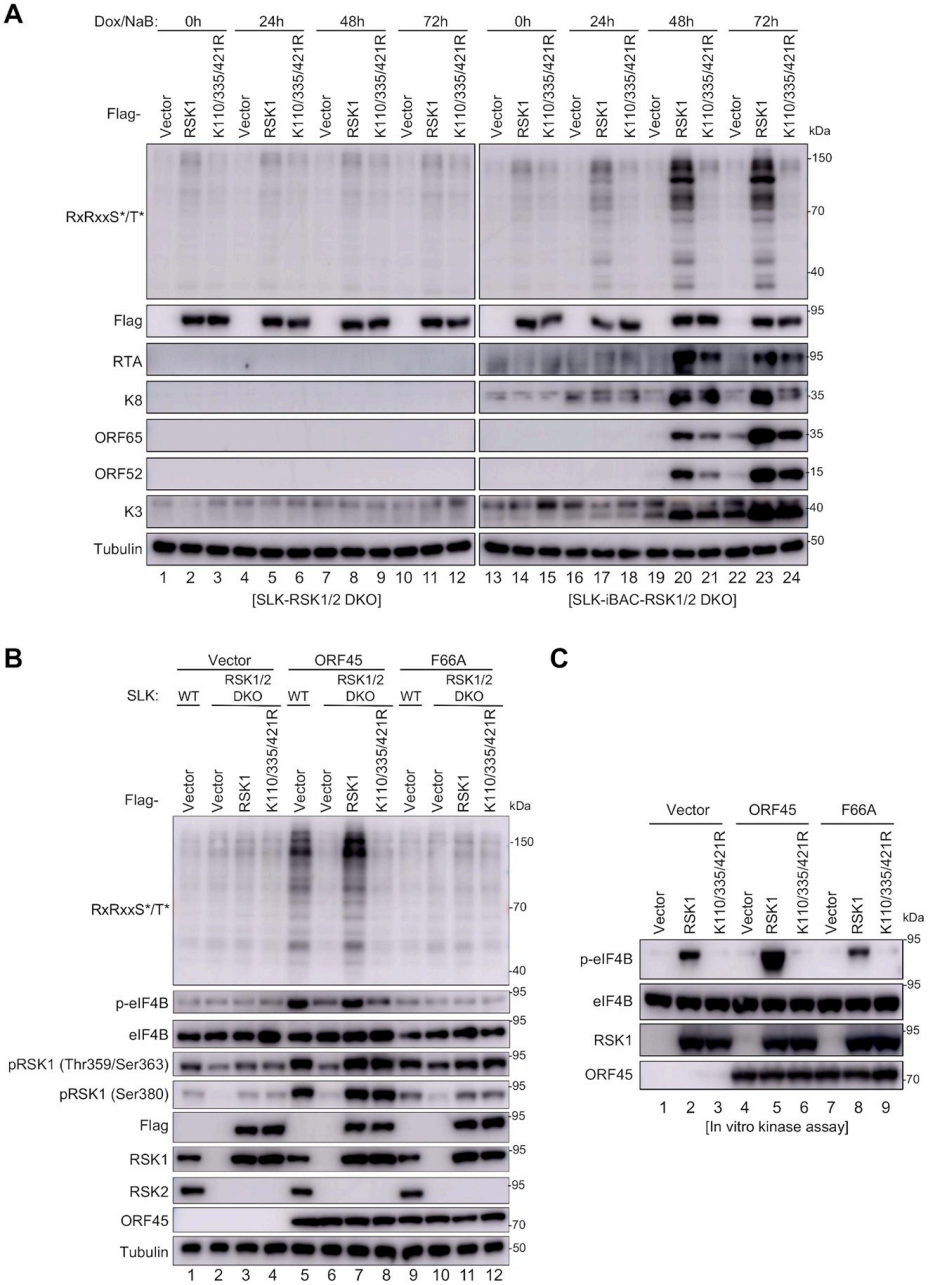
RSK1 SUMOylation affects the phosphorylation of its downstream substrates. (**A**) SUMOylation of RSK1 contributes to the overall phosphorylation of its substrates during KSHV lytic replication. SLK^RSK1/2 DKO^-vector, SLK^RSK1/2 DKO^-RSK1, SLK^RSK1/2 DKO^-RSK1^K110/335/421R^, SLK-iBAC^RSK1/2 DKO^-vector, SLK-iBAC^RSK1/2 DKO^-RSK1, or SLK-iBAC^RSK1/2 DKO^-RSK1^K110/335/421R^ cells were induced with doxycycline (2 μg/ml) and sodium butyrate (1 mM) and cell lysates were subjected to IB with indicated antibodies. (**B**) SUMOylation of RSK1 affects the overall phosphorylation of RSK1 substrates in the presence of KSHV ORF45. SLK-vector, SLK^RSK1/2 DKO^-vector, SLK^RSK1/2 DKO^-RSK1 and SLK^RSK1/2 DKO^-RSK1^K110/335/421R^ cells were infected with lentiviruses containing empty vector, ORF45^WT^ or ORF45^F66A^ as indicated. The cells were serum-starved for 24 h before sample collection and cell lysates were analyzed by IB with indicated antibodies at 48 h post-transfection. (**C**) SUMOylation site mutations affect eIF4B phosphorylation by RSK1 *in vitro*. RSK1 kinase complexes with or without ORF45 were purified from HEK293A cells transfected with indicated plasmid. HA-eIF4B was purified from transfected HEK293T cells by affinity purification. RSK1 (5 μl) were mixed with eIF4B (0.5 μg) in kinase assay buffer at 30 °C for 30 min. The reaction was stopped by adding 2 x SDS-PAGE loading buffer, followed by IB analysis with indicated antibodies.

Since SUMOylation does not alter RSK1 activation or kinase activity *in vitro* and SUMO modification is known to mediate protein-protein interaction or change subcellular localization [14], we hypothesized that RSK1 SUMOylation may affect its substrate phosphorylation. To test this hypothesis, we evaluated the overall phosphorylation level of RSK1 substrate in cells by anti-AGC substrate motif antibody (RxRxxS*/T*: R, arginine; S, serine; T, threonine; and x, any amino acid), which is the consensus phosphorylation motif by members of the AGC kinase family, such as RSK, AKT, and p70 S6 kinase (S6K) [5]. KSHV lytic replication promotes the overall phosphorylation at RxRxxS*/T* motif in SLK-iBAC cells through RSK1 and RSK2 [19]. In SLK-iBAC^RSK1/2 DKO^-RSK1^WT^ cells, KSHV lytic replication elevated the overall phosphorylation level at RxRxxS*/T* motif, which was much lower in SLK-iBAC^RSK1/2 DKO^-RSK1^K110/335/421R^ cells (Fig 5A, comparing lane 20 to lane 21, lane 23 to lane 24), indicating SUMOylation of RSK1 contributes to the overall phosphorylation of its substrates during KSHV lytic replication.

KSHV ORF45 specifically interacts with RSK1/2 and activates RSKs via forming ERK-RSK-ORF45 complexes [4,5], while F66A mutation on ORF45 disrupts ORF45-RSK interaction and cannot activate RSKs [3]. Therefore, we utilized KSHV ORF45^WT^ or ORF45^F66A^ to specifically stimulate RSK1 and examined the overall RxRxxS*/T* phosphorylation level in SLK^RSK1/2 DKO^-vector, SLK^RSK1/2 DKO^-RSK1^WT^, and SLK^RSK1/2 DKO^-RSK1^K110/335/421R^ stable cells. Compared to SLK^RSK1/2 DKO^-vector cells, KSHV ORF45^WT^ induced overall phosphorylation at the RxRxxS*/T* motif in SLK^RSK1/2 DKO^-RSK1 cells, but ORF45^F66A^ failed to induce (Fig 5B), indicating the increased phosphorylation of these substrates are ORF45-RSK dependent. In SLK^RSK1/2 DKO^-RSK1^K110/335/421R^ cells, the overall phosphorylation level at the RxRxxS*/T* motif was significantly lower upon ORF45^WT^ expression as compared to that in SLK^RSK1/2 DKO^-RSK1^WT^ cells (Fig 5B, comparing lane 8 to lane 7). The intensity of several immunoblotting bands was dramatically reduced in SLK^RSK1/2 DKO^-RSK1^K110/335/421R^ cells (Fig 5B, lane 8), indicating SUMO modification affects the phosphorylation of RSK1 substrates.

We have systematically identified the substrates of the ORF45-RSK-axis by evaluating the overall phosphorylation level at RxRxxS*/T* motif during KSHV lytic replication with immunoprecipitation using anti-RxRxxS*/T* motif specific antibody followed by quantitative mass spectrometry analysis [19]. The phosphorylation level at RxRxxS*/T* motif of several substrates increased more than 10-fold with ORF45^WT^ as compared to ORF45^F66A^, including eIF4B (Ser^422^ and Ser^425^, 29.91 fold), SETD1A (Ser^915^ and Ser^916^, 25.76 fold), LMNA (Ser^404^ and Ser^406^, 16.42 fold), ZNF217 (Ser^975^, 13.27 fold), SLBP (Ser^111^, 13.07 fold), RNF20 (Ser^517^, 12.49 fold), SP100 (Ser^221^, 12.46 fold), WHSC1 (Ser^172^, 12.36 fold), RNF19B (Ser^393^, 12.30 fold), TCOF1 (Ser^107^, 11.88 fold), DDI2 (Ser^194^, 11.24 fold), ZNF384 (Ser^234^, 11.02 fold), ZNF217 (Ser^974^, 10.91 fold), and DNAJC2 (Ser^47^, 10.69 fold) [19]. Due to the availability and quality of the commercial phosphorylation antibody of these substrates, we could only examine the phosphorylation of eIF4B in SLK^RSK1/2 DKO^-vector, SLK^RSK1/2 DKO^-RSK1^WT^, and SLK^RSK1/2 DKO^-RSK1^K110/335/421R^ stable cells with ORF45 stimulation. Consistently, KSHV ORF45^WT^ significantly induced eIF4B phosphorylation at Ser^422^ in SLK^RSK1/2 DKO^-RSK1^WT^ cell but not SLK^RSK1/2 DKO^-RSK1^K110/335/421R^ cells (Fig 5B), indicating RSK1 SUMOylation mediates the efficient phosphorylation of eIF4B. Consistently, wild-type RSK1 but not RSK1^K110/335/421R^ phosphorylated eIF4B *in vitro* and wild-type ORF45 but not ORF45^F66A^ promoted RSK1-mediated phosphorylation of eIF4B (Fig 5C). These results demonstrated that SUMOylation is required for the phosphorylation of some RSK1 substrates, such as eIF4B, which is required for efficient KSHV lytic replication.

### SUMO-SIM interaction is required for eIF4B phosphorylation by RSK1

**Fig 6:**
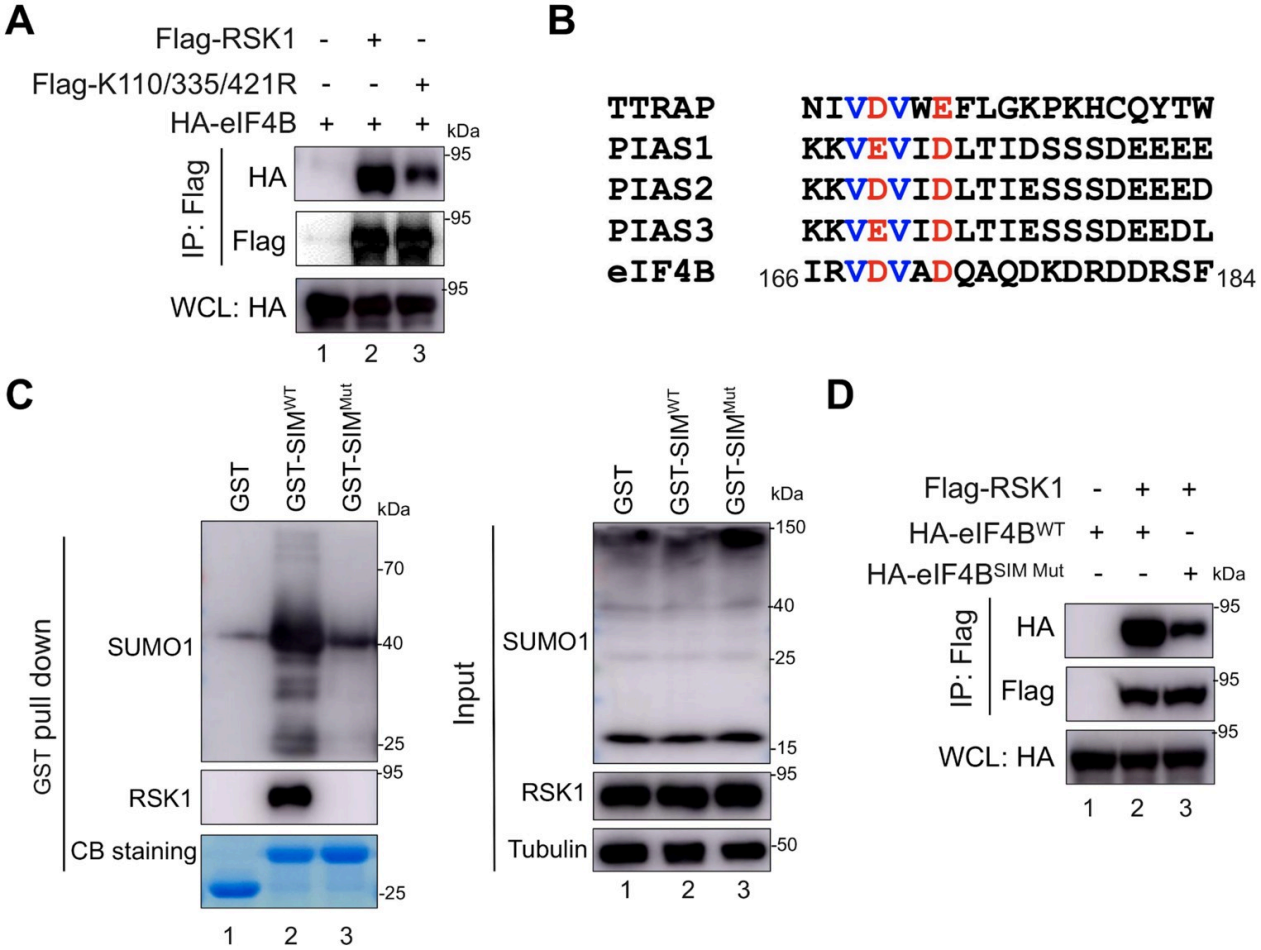
SUMO-SIM interaction contributes to RSK1-eIF4B association. (**A**) SUMOylation of RSK1 is required for efficient interaction between RSK1 and eIF4B. HEK293T cells were co-transfected with indicated plasmids and cell lysates were subjected to IP and IB with indicated antibodies at 48 h post-transfection. (**B**) Alignment of SIMs among eIF4B, PIAS1, PIAS2, PIAS3, and TTRAP. (**C**) eIF4B SIM binds to endogenous SUMO1 and RSK1. GST vector control and GST-tagged eIF4B SIM or its mutant were purified from *E*.*coli* and subjected to GST pull-down assay with HEK293T cell lysates. The co-precipitated proteins were analyzed by IB with indicated antibodies. (**D**) SIM mutation within eIF4B reduced the RSK1-eIF4B association. HEK293T cells were co-transfected with indicated plasmids and cell lysates were subjected to IP and IB with indicated antibodies at 48 h post-transfection.

RSK1 SUMOylation mediates eIF4B phosphorylation, which is required for efficient KSHV lytic replication [20]. We next investigated how SUMOylation is required for eIF4B phosphorylation. Due to the marginal effect of RSK1 kinase activation or activity by SUMOylation, we speculated that RSK1 SUMOylation may affect the kinase-substrate interaction. Indeed, RSK1^WT^ was associated with eIF4B, while RSK1^K110/335/421R^ interacted with eIF4B with less binding affinity than RSK1^WT^ (Fig 6A), suggesting SUMOylation of RSK1 is required for efficient interaction between RSK1 and eIF4B. SUMOylation modification could mediate protein-protein interaction through SUMO and SIM association, which is proposed as the Lys-Xaa_3-5_-[Val/Ile]-[Ile/Leu]_2_-Xaa_3_-[Asp/Glu/Gln/Asn]-[Asp/Glu]_2_ hydrophobic core motif, sometimes surrounded by Ser-Xaa-Ser motif [21]. Inspection the sequence of eIF4B revealed one potential SIM between the amino acid positions 166 and 170 (_166_IRVDV_170_) (Fig 6B). SIM^eIF4B^ fusion with GST tag readily bound to endogenous SUMO1 and RSK1, while mutations on GST-SIM^eIF4B^ abolished these interactions (Fig 6C). SIM mutation within eIF4B reduced the interaction between RSK1 and eIF4B (Fig 6D), suggesting that SUMO-SIM association contributes to RSK1-eIF4B interaction. These results demonstrated that RSK1 SUMOylation facilitates the interaction between RSK1 and eIF4B, leading to the efficient phosphorylation of eIF4B by the RSK1-ORF45 axis.

In conclusion, our results characterized the post-translational modification of RSK1 by SUMO, primarily at Lys^110^, Lys^335^, and Lys^421^, resulting in efficient phosphorylation of eIF4B, which is required for KSHV lytic replication.

## Discussion

KSHV modulates multiple cellular signaling pathways to favor virus replication [22]. SUMOylation has emerged as a major posttranslational modification of cellular and viral proteins, affecting a variety of cellular processes upon KSHV infection [23,24]. Multiple KSHV encoded proteins contain SIMs or are shown to be modified by SUMO to modulate the cellular SUMO network. For example, RTA contains tandem SIMs (SIM1: _273_MVDL_277_; SIM2: _285_AVIL_288_; and SIM3: _309_VSII_312_) and functions as a SUMO-targeting ubiquitin E3 ligase (STUbL) that prefers to bind and degrade the targets with poly-SUMO2/3 modifications [25]. The STUbL activity is required for RTA transactivation and KSHV lytic replication [25]. K8 is primarily SUMOylated at Lys^158^ and this SUMO modification is essential for the transcriptional repressive activity of K8 [26]. In addition to its role as a substrate for SUMO modification, K8 functions as a SIM-containing SUMO E3 ligase (SIM: _72_VIDL_75_) that shows higher specificity toward SUMO2/3 than SUMO1 [27]. LANA contains two SIMs (SIM1: _244_IYVG_247_; SIM2: _264_ISIG_267_) within the N-terminal domain that preferentially bind to SUMO2 and mediate the recruitment of chromatin remodeling proteins and transcriptional factors, such as TRIM28 and HIF-1α [28]. SUMOylation of Lys^1140^ of LANA is important for its function to maintain the KSHV episome and silence RTA activation during latency [28]. vIRF3 (also known as LANA2) has one SIM at the C-terminus and can be SUMOylated at multiple lysine residues. vIRF3 could inhibit SUMOylation of pRb, p107, and p130, leading to blockage of pRb-mediated cell growth arrest [29]. In addition, vIRF3 also disperses PML nuclear body by facilitating the SUMOylation and ubiquitination of PML in a SIM-dependent manner, which potentially contribute to KSHV-mediated cell transformation [30]. The SUMO modification or SUMO binding properties of these viral proteins dynastically control the establishment of latency or switch between latency and lytic replication, contributing to the production of progeny virus and pathogenesis of KSHV. We have shown here that RSK1 SUMOylation is dramatically increased during KSHV lytic replication, which in turn facilitates KSHV lytic replication with high efficiency. RTA, LANA, or K8 cannot promote RSK1 SUMOylation, suggesting they are not the SUMO E3 ligase for RSK1 (S2 Fig). How RSK1 is SUMOylated during KSHV lytic replication is still undetermined, and our group is actively working on this topic to identify the viral or cellular factors that control this process.

Posttranslational modifications modulate protein functions. Besides phosphorylation, other posttranslational modifications, such as ubiquitination and SUMOylation, also delicately regulate the activation and activity of AGC kinases. For example, AKT undergoes K63-linked ubiquitination by TRAF6, which is essential for the membrane recruitment and phosphorylation of AKT upon growth-factor stimulation [31]. AKT is also modified by SUMO at multiple lysine residues and mutations on these SUMOylation sites abolish AKT kinase activity, leading to impaired cell proliferation and tumorigenesis [17,32,33]. SUMOylation potentially changes the subcellular localization, kinase activity, protein stability, or binding partners of the target proteins [14]. Unlike AKT, SUMO modifications at Lys^110^, Lys^335^, and Lys^421^ do not alter RSK1 activation by upstream signals or kinase activity but affect the phosphorylation of its downstream substrates. Similar to phosphotyrosine-Src homology 2 (SH2) domain interaction-mediated selective protein-protein interaction (PPI) with phosphorylated tyrosine, SIM-SUMO interaction mediates selective PPI with SUMO modified proteins [14]. The SIM of eIF4B could serve as a docking site for SUMO association and mediates the interaction between eIF4B and RSK1, leading to efficient eIF4B phosphorylation by RSK1, which is required for KSHV lytic replication [20]. Although RSK isoforms (RSK1-RSK4) share 70–85% identity, only RSK1 and RSK2 undergo SUMOylation in normal condition, and SUMOylation of RSK1 is much robust than RSK2. Sequence alignment indicates that Lys^110^ is conserved among all RSK isoforms, Lys^335^ is only conserved between RSK1 and RSK3, and Lys^421^ is unique for RSK1, suggesting a unique role of SUMO modification of RSK1. Although RSK2-RSK4 have less or no modification by SUMO in normal conditions, their SUMOylation upon specific stimulations still needs to be further explored. Our results demonstrate that RSK1 SUMOylation is required for efficient KSHV lytic replication by at least regulating the phosphorylation of eIF4B. Because of its regulatory roles of eIF4B in regulating translation of a subset of mRNAs, RSK1 SUMOylation is expected to delicately modulate the cellular translation processes under certain conditions. In KSHV-infected cells, ORF45 contributes to sustained activation of RSKs by forming high molecular mass protein complexes with RSK and ERK to prevent their dephosphorylation, leading to efficient lytic replication of KSHV. Further studies are required to explore the physiological role of RSK1 SUMOylation in the absence of viral infection.

## Methods

### Antibodies and chemicals

Anti-RxRxxS*/T*(#10001S), RSK1 (#9333S), RSK2 (#5528S), p-eIF4B (#3591S), eIF4B (#3592S) antibodies were ordered from Cell Signaling Technology. HRP anti-HA (#901519), HRP anti-Flag (#637311) antibodies were purchased from BioLegend. pRSK1 (Thr359/Ser363) (#AP0539), pRSK1 (Ser380) (#AP1147), RSK3 (#A16305), ERK1/2 (#A10613), and SUMO1 (#A19121) were purchased from ABclonal. Anti-RTA (ORF50) monoclonal mouse antibody was given by Dr. Ke Lan (Wuhan University, China) [34]. Anti-ORF65 monoclonal mouse antibody was given by Dr. Shou-Jiang Gao (University of Pittsburgh, USA) [35]. Monoclonal antibodies against ORF45 and ORF52 and polyclonal antibodies against K3, K8, and ORF55 were described previously [36–38]. EZview Red ANTI-FLAG M2 Affinity Gel (#F2426) and sodium butyrate (#B5887) were ordered from Sigma, Doxycycline (#S4163) was ordered from Selleck, Glutathione Sepharose 4B (#17-0756-01) were ordered from GE Healthcare. ClonExpress II One Step Cloning Kit (#C122-01) and HiScript II Q RT SuperMix for qPCR (+gDNA wiper) (#R223-01) were purchased from Vazyme Biotech. Lipofectamine 3000 (#3000015) were purchased from Thermo Fisher Scientific.

### Cells

BJAB, A549, HeLa, SLK, HEK293T, and HEK293A cells were cultured in Dulbecco’s modified Eagle’s medium (DMEM) containing 10% FBS and antibiotics. BCBL1 cells were given by Dr. Ersheng Kuang (Sun Yat-Sen University, China) and were cultured in RPMI 1640 containing 20% FBS and antibiotics. SLK-iBAC were generated as previously described and cultured in DMEM containing 10% FBS, antibiotics, and hygromycin (400 μg/ml) [19]. iSLK-BAC16 cells were cultured in DMEM containing 10% FBS, antibiotics, puromycin (1 μg/ml), G418 (250 μg/ml), and hygromycin (400 μg/ml). Doxycycline (2 μg/ml) and sodium butyrate (1 mM) treatment was used to induce KSHV lytic replication in SLK-iBAC or iSLK-BAC16 cells.

### Plasmid constructs

The cDNA for human RSK1 and eIF4B were obtained from the Core Facility of Basic Medical Sciences, Shanghai Jiao Tong University School of Medicine. pCR3.1-ORF45 is originally cloned by Dr. Fanxiu Zhu (Florida State University, USA). The mutations and internal deletion mutants of ORF45 were generated using ClonExpress II One Step Cloning Kit (Vazyme Biotech, #C122-01). Guide RNAs (gRNAs) targeting RSK1 and RSK2 were cloned into LentiCRISPR V2-Puro (Addgene #52961) and lentiCRISPR v2-Blast (Addgene, #112233), respectively. gRNA sequences were described previously [19]: RSK1-Top: CACCGAGCCTTGACGTGGTGCGTGA, RSK1-Bottom: AAACTCACGCACCACGTCAAGGCTC, RSK2-Top: CACCGTCACCTCAGCGCTGTCGGAC, RSK1-Bottom: AAACGTCCGACAGCGCTGAGGTGAC. The gRNA targeting sequences of RSK1^WT^ and RSK1^K110/335/421R^ were synonymously mutated to avoid the gene silencing mediated by gRNAs. RSK1^WT^, RSK1^K110/335/421R^, eIF4B, eIF4B^WT^, eIF4B^3A^, ORF45^WT^, ORF45^F66A^, SUMO1, SUMO1^G/A^, SUMO2, SUMO2^G/A^, RSK1^NT^ and RSK1^CT^ and their mutants were subcloned into pKH3, pLVX-3Flag, or pCDH-3Flag vectors as indicated. S6 peptide (KEAKEKRQEQIAKRRRLSSLRASTSKSESSQK), eIF4B SIM^WT^ peptide (LSALSLNEESLGNRRIRVDVADQAQDKDRDDRSFG) and eIF4B SIM^Mut^ peptide (LSALSLNEESLGNRRARADAADQAQDKDRDDRSFG) were cloned into pGEX-4T vector. All constructs were sequenced using an ABI PRISM 377 automatic DNA sequencer to verify 100% correspondence with the original sequence.

### Generation of stable cell lines

For stable expression, lentiviral plasmids harboring the desired genes were co-transfected into HEK293T cells with the packing plasmids pSPAX and pMD2G at a ratio of 5:3:2. The supernatants containing lentiviruses were collected at 48 h post-transfection and used to infect indicated cells in the presence of polybrene (8 μg/ml). Stable expression cells were selected by puromycin (2 μg/ml), hygromycin (400 μg/ml), or blasticidin (10 μg/ml) at 48 h post-infection until all the control cells were killed. For generation of knockout cells by the CRISPR/Cas9 system, single clones were isolated and assayed by western blot analysis for RSK1/RSK2 protein expression.

### *In vitro* SUMOylation assay

SUMO1 (BostonBiochem, #K-700), SAE1/UBA2 (BostonBiochem, # E1-315), and ubc9 recombinant proteins (BostonBiochem, #E2-645), SUMO conjugation reaction buffer (BostonBiochem, #SK-15), and ATP/Mg (BostonBiochem, #SK-15) were purchased from Boston Biochem. Flag-tagged RSK1 was purified from transfected HEK293T cells by affinity purification via anti-Flag M2 affinity Gel (Sigma, #F2426). 1 μg purified RSK1 substrate was mixed with 2 μg SUMO1, 200 ng SAE1/UBA2, 100 ng ubc9, 1 x ATP/Mg, and 1 x SUMO reaction buffer in 20 μl reaction system for 3 h at 30°C and stopped by adding stop buffer (BostonBiochem, #SK-15). The reaction mixture was subjected to standard immunoblotting analyses to detect the SUMOylation of RSK1.

### Immunoprecipitation and immunoblotting

For co-immunoprecipitation, 2×10^6^ HEK293T cells were transfected with 20 μg of plasmid at a confluency of 90% with Lipofectamine 3000 (Thermo Fisher Scientific, #3000015). The cells were washed twice with cold phosphate-buffered saline (PBS) and lysed in a whole cell lysis buffer (WCL) containing (50 mM Tris·HCl [pH 7.4], 150 mM NaCl, 1% NP-40, 1 mM EDTA, 10% glycerol, protease inhibitor cocktail [Roche]) for 20 min on ice at 48 h post-transfection. The cell lysates were then centrifuged at 15,000 rpm for 15 min and the clear supernatants were subjected to immunoprecipitation with anti-Flag M2 agarose resin (Sigma, #F2426) following the manufacturer’s instruction. After 4h incubation at 4°C, the beads were washed for three times with WCL and twice with PBS, and then boiled with the 2 x SDS loading buffer for 10 min. The immunoprecipitants were applied to standard immunoblotting analyses with specific antibodies.

Immunoprecipitation in denaturing conditions for detecting SUMOylated protein was described previously [39]. Briefly, cells were lysed in SDS lysis buffer made by 1:3 ratio of Buffer I (5% SDS, 0.15 M Tris-HCl pH 6.8, 30% glycerol) and Buffer II (25 mM Tris-HCl pH 8.3, 50 mM NaCl, 0.5% NP-40, 0.5% deoxycolate, 0.1% SDS, 1 mM EDTA) supplemented with protease inhibitors cocktail (Roche), 1 mM DTT and 5 mM N-Ethylmaleimide (NEM, Sigma) and denatured 5 min at 95°C. Cell lysates were then centrifuged at maximum speed for 10 min. Supernatants were either directly resolved by SDS-PAGE or diluted 1:5 in E1A buffer (50 mM Hepes pH 7.5, 250 mM NaCl, 0.1% NP-40, 1 mM EDTA, supplemented with protease inhibitors cocktail, 1 mM DTT and 5 mM NEM) and then immunoprecipitated using anti-Flag M2 agarose resin (Sigma, #F2426). After 4 h incubation at 4°C, the beads were washed for three times with WCL and twice with PBS, and then boiled with the 2 x SDS loading buffer for 10 min. The immunoprecipitants were applied to standard immunoblotting analyses with specific antibodies.

### Recombinant protein expression and purification

S6 peptide, eIF4B SIM^WT^ peptide and eIF4B SIM^Mut^ peptide were cloned into pGEX-4T vector with a N-terminal GST tag. The transformed *E*. *coli* were cultured in LB medium with appropriate antibiotic until OD600 reached 0.7. Protein expressions were induced 4 hours at 37°C with 1 mM isopropyl b-D-1 thiogalactopyranoside (IPTG). Cells were washed and resuspended in PBS buffer, followed by sonication, and then the cell debris was removed by centrifugation (15,000 rpm, 4°C, 15 min). The proteins with GST tag in the supernatant were affinity-purified by Glutathione Sepharose 4B (GE Healthcare, #17-0756-01).

### RNA purification and RT-qPCR

Total RNA was extracted from cells with TRIzol reagent (Sigma) according to the manufacturer’s protocol. Afterwards 0.5 μg of total RNA was reverse transcribed by HiScript II Q RT SuperMix for qPCR (+gDNA wiper) (Vazyme Biotech, #R223-01) and the cDNA was quantified by SYBR green based qPCR using gene specific primers. The relative level of gene expression was calculated by the 2^-ΔCt^ and the ΔΔCt methods, where GAPDH was used for normalization. The RT-qPCR graphs represent the average of at least three independent experiments. The sequences of the primers used in RT-qPCR have been listed in S1 Table.

### Measurement of viral DNA copy number during KSHV lytic replication

SLK-iBAC-vector, SLK-iBAC^RSK1/2 DKO^-vector, SLK-iBAC^RSK1/2 DKO^-RSK1 and SLK-iBAC^RSK1/2 DKO^-RSK1^K110/335/421R^ cells were uninduced or induced with doxycycline (2 μg/ml) and sodium butyrate (1 mM), and lysed in RIPA buffer followed by sonication and then centrifugation to remove cell debris. Total DNA was purified from the supernatant by phenol-chloroform extraction and 10 ng of total DNA was analyzed in qPCR. The viral DNA was measured by qPCR using primers for ORF11 (Fw-GGCACCATACAGCTTCTACGA and Rev-CGTTTACTACTGCACACTGCA). The amount of viral DNA was normalized for the cellular DNA input, which was measured by qPCR specific for the β-actin genomic region (Fw-CGGGAAATCGTGCGTGACATT; Rev-CAGGAAGGAAGGCTGGAAGAGTG).

### Quantification of extracellular virion genomic DNA by real-time qPCR

SLK-iBAC-vector, SLK-iBAC^RSK1/2 DKO^-vector, SLK-iBAC^RSK1/2 DKO^-RSK1 and SLK-iBAC^RSK1/2 DKO^-RSK1^K110/335/421R^ cells were uninduced or induced with doxycycline (2 μg/ml) and sodium butyrate (1 mM), and viral DNA was isolated from supernatant medium as previously described [40]. Briefly, medium from the infected cells was centrifuged to remove any cellular debris and treated with TurboDNase (Ambion) to remove any unprotected DNA. The viral particles were lysed with buffer AL (Qiagen), and the proteins were degraded with protease K (Qiagen). The DNA was then extracted using phenol-chloroform extraction and analyzed by SYBR green real-time PCRs with KSHV-specific primers ORF11 described above. Viral DNA copy numbers were calculated with external standards of known concentrations of serially diluted BAC16 DNA ranging from 1 to 10^7^ genome copies per reaction.

### *In Vitro* kinase assay

*In vitro* kinase assay with GST-S6 peptide (_218_KEAKEKRQEQIAKRRRLSSLRASTSKSESSQK_249_) and RSK1 was described previously [20]. Briefly, HEK293A cells were co-transfected with RSK or RSK^K110/335/421R^ and ORF45 or ORF45^F66A^ as indicated. At 24 h post-transfection, the cells were serum-starved for an additional 24 h. WCL were collected and subjected to immunoprecipitation with 50 μl of anti-Flag affinity beads to purify RSK1 kinase complexes at 48 h post-transfection. After two washes with the lysis buffer and three washes with TBS buffer (50 mM Tris-HCl, pH 7.4, 150 mM NaCl), the immunoprecipitated beads were resuspended in 100 μl of TBS plus 1 mM PMSF, 1 mM Na_3_VO_4_, 1×protease inhibitor mixture (Roche). The kinase reaction was performed by incubation of 5 μl of the precipitated RSK1 complexes with 2.5 μg of GST-S6 substrate in 25 μl of 1×kinase assay buffer (25 mM HEPES, pH 7.5, 50 mM NaCl, 20 mM -glycerophosphate, 1 mM DTT, 20 mM MgCl_2_,1 mM Na_3_VO_4_, 1 μg/ml BSA, 20 μM ATP). The reactions were kept at 30 °C for 30 min and stopped by addition of 2 × SDS- loading buffer. The samples were analyzed by immunoblotting with the RxRxxS*/T* antibody to evaluate the phosphorylation level of S6 peptide.

### GST pull-down assay

HEK293T cells were transfected with pKH3-SUMO1 or pKH3-SUMO2. At 48 h post-transfection, cells were harvested and lysed in a buffer containing 20 mM Tris-HCl at pH 7.5, 0.5% NP-40, 150 mM NaCl and protease inhibitors. The GST-tagged wild-type or mutant eIF4B SIM or GST control proteins were purified by Glutathione Sepharose 4B (GE Healthcare, #17-0756-01), followed by incubation of WCL from cells transiently expressing SUMO1 or SUMO2 for 2h at 4 °C. After washing three times with lysis buffer and twice with PBS, the beads were heated in 2 × SDS loading sample buffer at 95 °C for 10 minutes and the coprecipitated proteins were analyzed by immunoblotting with indicated antibodies.

### Quantification and statistical analysis

All data were expressed as mean ± SD, unless otherwise noted. For parametric analysis, the F test was used to determine the equality of variances between the groups compared; statistical significance across two groups was tested by Student’s t-test; one-way analysis of variance (ANOVA) followed by Bonferroni’s *post hoc* test were used to determine statistically significant differences between multiple groups. *P*-values of less than 0.05 were considered significant.

## Supporting information

S1 Fig Map of KSHV iBAC. (TIF)

S2 Fig RTA, LANA, and K8 do not affect RSK1 SUMOylation. HEK293T cells were co-transfected with indicated plasmids and cell lysates were subjected to IP and IB with indicated antibodies.
(TIF)

S1 Table List of primer sequences for qRT-PCR array. (DOCX)
