# Supplementary figures and images for: RSK1 SUMOylation is required for KSHV lytic replication
Source: PLoS Pathog. 2021 Dec 6;17(12):e1010123. doi: 10.1371/journal.ppat.1010123 (PMC8675914; doi:10.1371/journal.ppat.1010123)

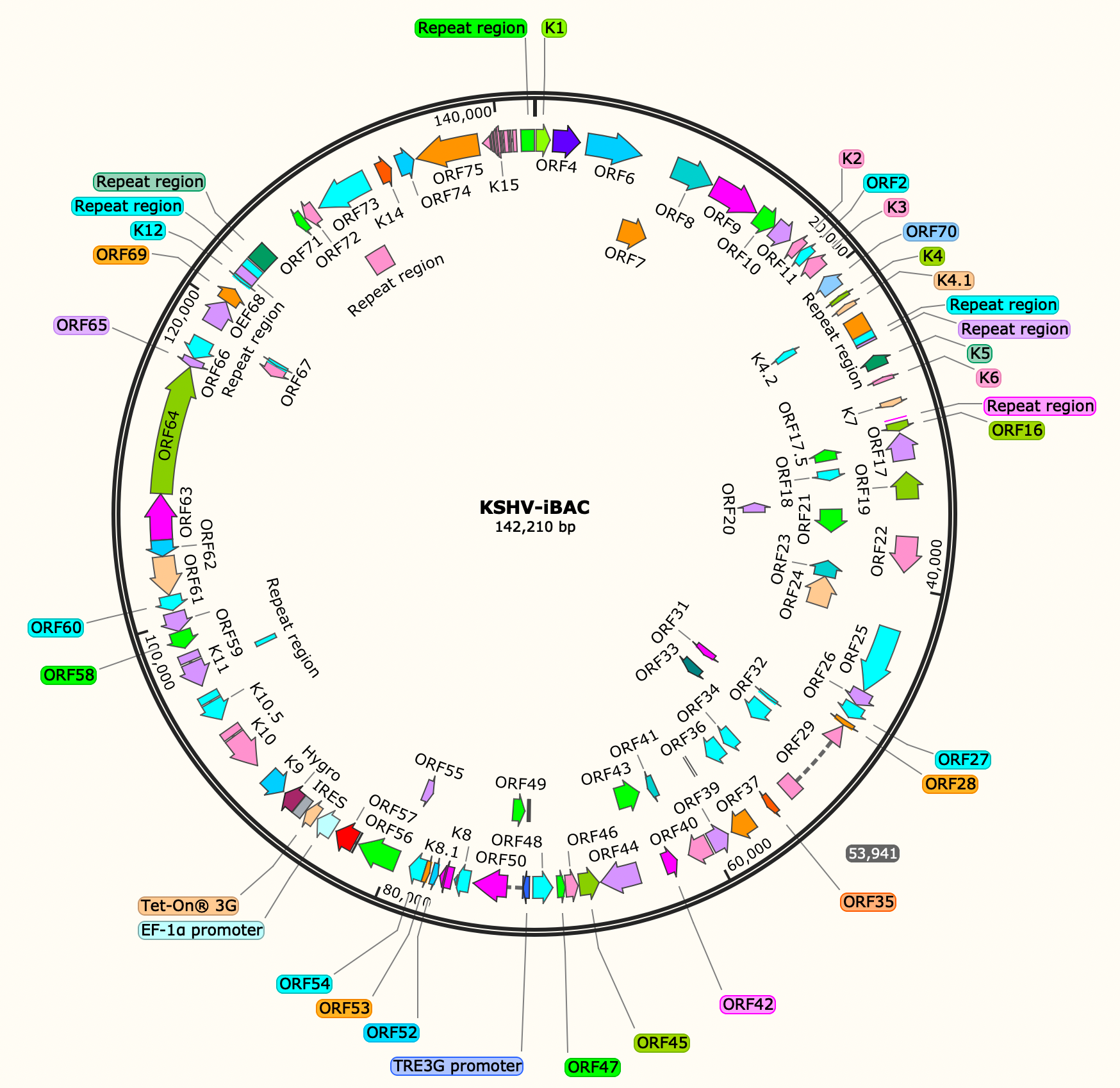

Supplement: S1 Fig — (TIF) [file ppat.1010123.s001.tif]

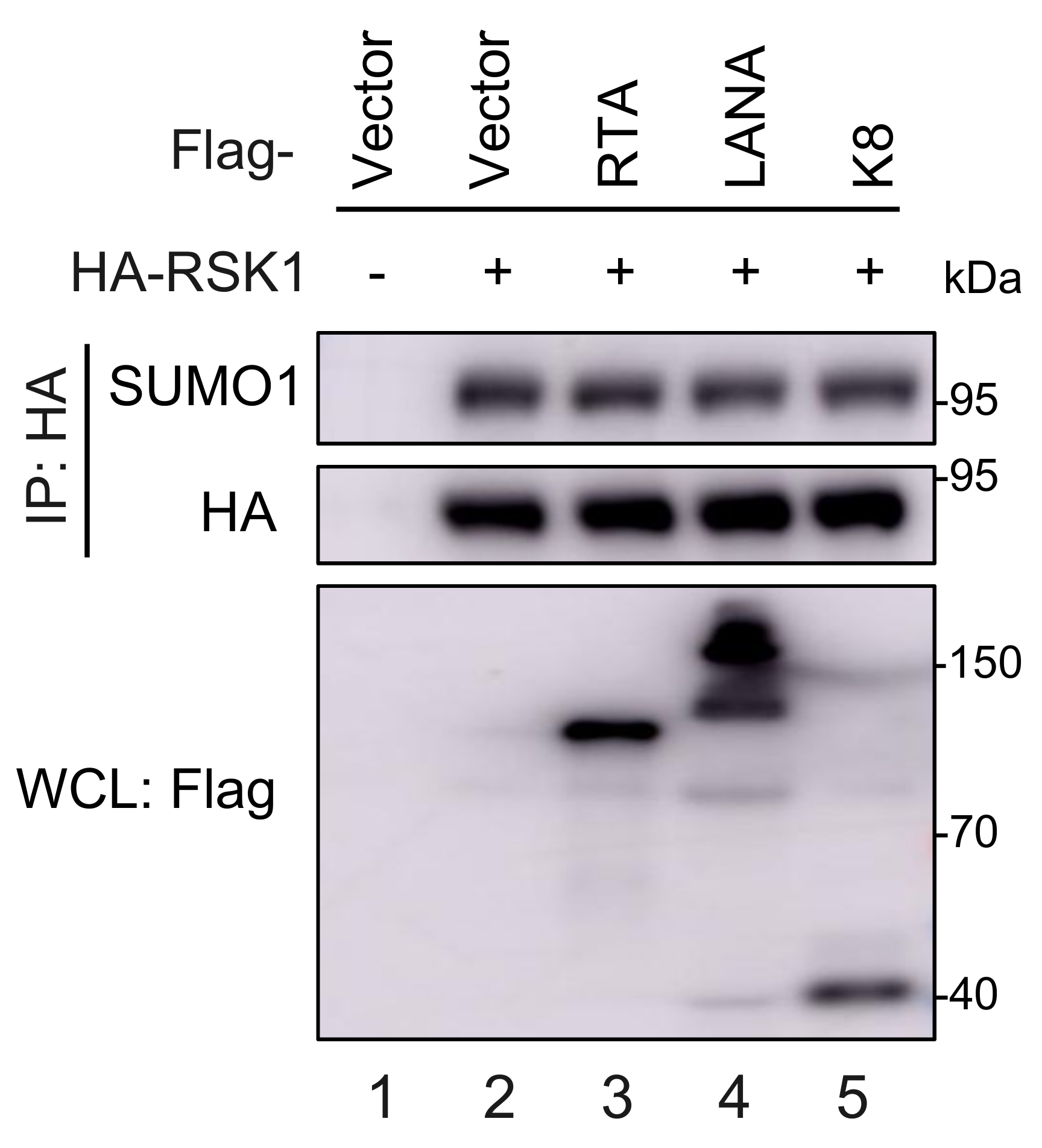

Supplement: S2 Fig — HEK293T cells were co-transfected with indicated plasmids and cell lysates were subjected to IP and IB with indicated antibodies. (TIF) [file ppat.1010123.s002.tif]
