# Supplementary material for: RSK1 SUMOylation is required for KSHV lytic replication
Source: PLoS Pathog. 2021 Dec 6;17(12):e1010123. doi: 10.1371/journal.ppat.1010123 (PMC8675914; doi:10.1371/journal.ppat.1010123)
Supplement: S1 Table — (DOCX) [file ppat.1010123.s003.docx]

S1 Table

KSHV primers used for reverse-transcription real-time quantitative PCR

| Primer pair | Gene location (KSU75698) | Orientation (splicing) | Target position (KSU75698) | Product size | Forward primer sequence | Reverse primer sequence |
| --- | --- | --- | --- | --- | --- | --- |
| K1 | 105–974 | + | 196–280 | 85 | TGATTTCAACGCCTTACACG | CGCAAAAGCCGAGTATTGTT |
| ORF4 | 1142–2794 | + | 2638–2702 | 65 | GCCTCAGAGACCGCGAGA | AGCGATTTTTAGACGCCGG |
| ORF6 | 3210–6611 | + | 5302–5367 | 66 | CTGCCATAGGAGGGATGTTTG | CCATGAGCATTGCTCTGGCT |
| ORF7 | 6628–8715 | + | 8259–8322 | 64 | TTTATTTCCCAGTCCTCCAAATG | GGGAAGCATGCCCGC |
| ORF8 | 8699–11236 | + | 11003–11065 | 63 | CCCGACGTAGATCGCAGG | GTTTTTGATTTCCTCCCGTGTT |
| ORF9 | 11363–14401 | + | 12070–12131 | 62 | TAGGCGCTTCGTGCTGG | CCGGATTGCTGCACTCGTA |
| ORF10 | 14519–15775 | + | 15583–15645 | 63 | GGGCGTGGCAATGGC | AAGCTGTATGGTGCCTGGCT |
| K2 | 17875–17261 | - | 17754–17690 | 65 | ACCCTTGCAGATGCCGG | GGATGCTATGGGTGATCGATG |
| ORF2 | 18553–17921 | - | 18119–18057 | 63 | TGCTCGCCAGGCTTGG | CGTGTTTCTCTCGCATGATAGC |
| K3 | 19609–18608 | -(spl) | 18809–18749 | 61 | AGCCCCATCGCCCG | TGAGCGGTATAGGGCCACTTAC |
| ORF70 | 21104–20091 | - | 20257–20197 | 61 | AGGCGCGGAAAGGGAC | AAACGCATATAGAGCCACTACGG |
| K4 | 21832–21548 | - | 21795–21735 | 61 | TTGTCCGGTCTATGCCAGG | CTGCCTTGCTTTGTTTGCAA |
| K5 | 26483–25713 | -(spl) | 25987–25924 | 64 | ACAAGGACCGTCAATTCGATG | TGCCATACCGACGGCC |
| K6 | 27424–27137 | - | 27393–27327 | 67 | GGCGTGTACGACACGAGTGA | GCGTACTGCTTGCCACGTT |
| ORF17 | 32482–30821 | - | 30942–30879 | 64 | GAGCGACTGCTGGCTTCAAC | CGGTGGAGAAAGACGCTCC |
| ORF18 | 32424–33197 | + | 33113–33175 | 63 | AACGTATGCGGTCTCGGGT | GCACCAAGGTAGGCCAGCT |
| ORF19 | 34843–33194 | - | 33734–33673 | 62 | ATACCAGGTTCAAGCGGCG | TGGATTGCTGGAGTTTGGG |
| ORF20 | 35573–34611 | - | 35296–35233 | 64 | CGGCTACTTAGAAACCGCCA | CCACCTACCGCCGGC |
| ORF21 | 35383–37125 | + | 36960–37022 | 63 | CGTAGCCGACGCGGATAA | TGCCTGTAGATTTCGGTCCAC |
| ORF22 | 37113–39305 | + | 38129–38195 | 67 | TCGGCAGTATGCGGAACTG | AGTGGTGAACGTGGGCATG |
| ORF23 | 40516–39302 | - | 39467–39403 | 65 | TGCCGTCACATATCAGTTCGA | CCCCAAAGACCGTCAAAGC |
| ORF24 | 42778–40520 | - | 40890–40825 | 66 | AGAAGTCAAACAGGCCCCG | GTTCGTTTCTCAGGCTTGACG |
| ORF26 | 46933–47850 | + | 47287–47519 | 233 | AGCCGAAAGGATTCCACCAT | TCCGTGTTGTCTACGTCCAG |
| ORF27 | 47873–48745 | + | 48313–48375 | 63 | CACCACGTTTGGACGCATT | TAATCCGTAGGCCTGCCGT |
| ORF28 | 48991–49299 | + | 49021–49083 | 63 | GGAGGAATGGTGGACGGC | AAGACCAATCACGGGAGGCT |
| ORF29b | 50417–49362 | -(spl) | 50034–49917 | 118 | GAAGTGCCTTGGAAAACAGC | GCTTCTGGTGGGAGTCTGAG |
| ORF30 | 50623–50856 | + | 50707–50773 | 67 | GAGCAAGTGGTCGCGGG | TTTTGTGACATAGAGAGTCAGCGAG |
| ORF31 | 50763–51437 | + | 51125–51217 | 93 | TGTGCGGTATTTGCAGACAT | ATAATGGCCGAGATGGTGTC |
| ORF32 | 51404–52768 | + | 52636–52698 | 63 | GAGTCTTGTGGCATGCGTGA | CCCCCAGGTAACACAAGCC |
| ORF33 | 52761–5369 | + | 953492–53579 | 88 | GACCGGGAATGGAGTGACTA | AGCTGTTACCCTGCTCTGGA |
| ORF29a | 54676–53738 | - | 54302–54214 | 70 | GGCCAGAAAAACACACGACT | CGTTCAGAAAGGACGAAAGG |
| ORF34 | 54675–55658 | + | 55135–55196 | 62 | ACCCCCTTCCGTTGCTATG | ACAGTCGGCCCGACAAAA |
| ORF35 | 55639–56091 | + | 55838–55894 | 57 | AGGCGGGCCAGAGGTTT | GCGGCTGGCGCAAA |
| ORF37 | 57273–58733 | + | 58593–58661 | 69 | CCCGTCTACTTTCCCCGAG | ACTTCTTGACCAAAAGTTGGCAG |
| ORF38 | 58688–58873 | + | 58743–58811 | 69 | GGGAACCGCTCGACGTAGT | GCTCAAGCAACATGCCCTTT |
| ORF39 | 60175–58976 | - | 59145–59083 | 63 | TGGTCTTTGCTGGGAGGG | CGCCGACGGTCGATAGAA |
| ORF40 | 60308–61681 | + | 60665–60780 | 116 | AACGTCAGAACACCCAGACC | ATAGAGCTGTGCCACGTTCC |
| ORF41 | 61827–62444 | + | 61986–62090 | 105 | GGACCAGACACTGAGGGAAA | GTTTAGGGCTCGTTCAATGC |
| ORF42 | 63272–62436 | - | 62837–62778 | 60 | GACGAAGGCCGCGTCC | ATTATTTGTCGCGCCAGAAAG |
| ORF43 | 64953–63136 | - | 63520–63454 | 67 | GGATATGGTGTCCTGAGAATAGGTG | GCTGGCTCCCGTTGTTGA |
| ORF44 | 64892–67258 | + | 66469–66533 | 65 | GCCGGTGTCTCAAGAGCTG | TGTCCCCCTCCTGCCC |
| ORF45 | 68576–67353 | - | 67668–67606 | 63 | GCTTTGCGGCTTAAGTTTGG | CGCCTCCTCTGGTAGCGA |
| ORF46 | 69404–68637 | - | 68916–68813 | 104 | CTGGGATTGGTTCACGAGTT | TGAGCGGAGTTCTGTCAATG |
| ORF47 | 69915–69412 | - | 69486–69424 | 63 | TTGACCTGCGTGCGCTC | GGTTCTGTTAGCGGAAGTCAGAC |
| ORF49 | 72538–71630 | - | 72142–72081 | 62 | ACAAAATGGGAGAGGCACCA | GCGCCCCTGGAATCAGA |
| K8 | 74850–75569 | +(spl) | 75728–75795 | 68 | CATGCTGATGCGAATGTGC | AGCTTCAACATGGTGGGAGTG |
| K8.1 | 76433–76714 | +(spl) | 76509–76583 | 75 | TGGTGCTAGTAACCGTGTGCC | TCTGCATTGTAGTGCGCGTC |
| ORF52 | 77197–76802 | - | 76915–76856 | 60 | GGCACCAGGAGGCGGT | TCGCTTAGAATCGACGTCTGC |
| ORF53 | 77665–77333 | - | 77543–77481 | 63 | GCAACGTCATAGAATCCTGGG | GCTCAGCGCCAGGCCT |
| ORF54 | 77667–78623 | + | 78444–78505 | 62 | TTGCGCCATAGGAAGCTAGC | TCGCGAAAATGCACTCGAG |
| ORF55 | 79448–78765 | - | 78889–78830 | 60 | ACGAATGCATCGCGGAA | CGGAGGCAACTTTACCCAAG |
| ORF56 | 79436–81967 | + | 80735–80851 | 117 | GACGGCCTAGAGCGATACTG | CGATAGGCTGAGGTCATGGT |
| K9 | 85209–83860 | - | 85347–85207 | 141 | GGCCCACTAATATGTCAGCCA | CATTGTCCCGCAACCAGACT |
| K10 | 88164–86074 | - | 86577–86512 | 66 | CCCAACAGGCCAGCTACATAA | CTTCGTGGAACTCTGAGACGC |
| K10.5 | 91394–90936 | -(spl) | 91020–90942 | 79 | TGGTCTTCTCCGATGCTTCT | TCACCTACACAGTGGGTCATCAC |
| K11 | 93367–91964 | - | 92092–92018 | 75 | ATCCGAGTCATATTCAGGCGA | AATCGAGAACCTGAAGGGTCC |
| ORF58 | 95544–94471 | +(spl) | 95009–95121 | 113 | TGCGGAGCATTTATGGTGTA | TGCCTAAATGCCAAAAGTCC |
| ORF59 | 96739–95549 | - | 95813–95715 | 99 | CGAGTCTTCGCAAAAGGTTC | AAGGGACCAACTGGTGTGAG |
| ORF60 | 97787–96870 | - | 97246–97145 | 102 | GCCTTGCCAACGATTACATT | CGTGACTGGGTTTTTCCTGT |
| ORF61 | 100194–97816 | - | 97956–97891 | 66 | CCCATCTTGTTTCATCCCAGA | CTGACGGCTCTTCAGTGCC |
| ORF62 | 101194–100199 | - | 100283–100220 | 64 | GCCACACGCGGCCTC | TCTGAACGTGAAGGGCACG |
| ORF63 | 101208–103994 | + | 103279–103340 | 62 | GCGACTTCGTGCGCGT | ATGCGACAGATGTACGTGCG |
| ORF65 | 112443–111931 | - | 112340–112244 | 97 | ATATGTCGCAGGCCGAATAC | CCACCCATCCTCCTCAGATA |
| ORF66 | 113759–112470 | - | 112558–112494 | 65 | GAACTCCAGCAGCTGTGAGGT | CTGCCCTATTAAAGCACCGTG |
| ORF67 | 114508–113693 | - | 114437–114346 | 92 | TCAGTCCCTGGATTTGGAAC | CGTGCTGCATTCTAACCGTA |
| ORF68 | 114768–116405 | + | 115770–115833 | 64 | GTGGTCGCATCCCACGA | ATGGACCCTGTGAGGTGTCTG |
| ORF69 | 116669–117346 | + | 117241–117302 | 62 | TGCAGTGCAGGTACACACCA | GCATCTCGTCGGTGCAGTCT |
| K12 | 118101–117919 | - | 118070–117990 | 81 | TTCATGTCCCGGATGTGTTA | TAATCGCCAACAGACAAACG |
| ORF71 | 122710–122291 | - | 122268–122156 | 113 | GGATGCCCTAATGTCAATGC | GGCGATAGTGTTGGGAGTGT |
| ORF72 | 123566–122793 | -(spl) | 122996–122936 | 61 | CATTGCCCGCCTCTATTATCA | ATGACGTTGGCAGGAACCA |
| K14 | 127884–128930 | +(spl) | 128830–128890 | 61 | TGGTGGGCCTATTTGGGATA | GATGCACCGCCCTGCTT |
| ORF75 | 134441–130551 | - | 131326–131257 | 70 | GAGAACCCCGACAAGGACTG | ACACGGGCTTTGAGGTGG |
| K15 | 136772–134762 | -(spl) | 404–293 | 112 | CCCAATGTATTCGGGTATGC | TCCCACCATCAACCCTTAAA |
| H-GAPDH |  |  |  |  | GAAGGTGAAGGTCGGAGTC | GAAGATGGTGATGGGATTTC |
